# A Schedule-based Multi-channel MAC Protocol for Wireless Sensor Networks

**DOI:** 10.3390/s101009466

**Published:** 2010-10-21

**Authors:** Md. Abdul Hamid, M. Abdullah-Al-Wadud, Ilyoung Chong

**Affiliations:** 1 Department of Information & Communications Engineering, Hankuk University of Foreign Studies, 89 Wangsan-ri, Mohyun-myon, Cheoin-Gu, Yongin-si, Kyonggi-do, Yongin, 449-791, Korea; E-Mail: iychong@hufs.ac.kr (I.L.); 2 Department of Industrial & Management Engineering, Hankuk University of Foreign Studies, 89 Wangsan-ri, Mohyun-myon, Cheoin-Gu, Yongin-si, Kyonggi-do, Yongin, 449-791, Korea; E-Mail: wadud@hufs.ac.kr (M.A.-A.-W)

**Keywords:** wireless sensor networks, medium access control, multi-channel MAC, multi-channel single radio, collision avoidance

## Abstract

Due to the half-duplex property of the sensor radio and the broadcast nature of wireless medium, limited bandwidth remains a pressing issue for wireless sensor networks (WSNs). The design of multi-channel MAC protocols has attracted the interest of many researchers as a cost effective solution to meet the higher bandwidth demand for the limited bandwidth in WSN. In this paper, we present a scheduled-based multi-channel MAC protocol to improve network performance. In our protocol, each receiving node selects (schedules) some timeslot(s), in which it may receive data from the intending sender(s). The timeslot selection is done in a conflict free manner, where a node avoids the slots that are already selected by others in its interference range. To minimize the conflicts during timeslot selection, we propose a unique solution by splitting the neighboring nodes into different groups, where nodes of a group may select the slots allocated to that group only. We demonstrate the effectiveness of our approach thorough simulations in terms of performance parameters such as aggregate throughput, packet delivery ratio, end-to-end delay, and energy consumption.

## 1. Introduction

Due to rapid technological advances, a certain geographical location can be visualized as a fully connected information space using fine granularity processing, which can be implemented using sensor technology. Sensor nodes may be regarded as atomic computing particles, which can be deployed to geographical locations for capturing and processing data of their surroundings. The expected achievement of such sensor networks is to produce, over an extended period of time, global information from local data sensed by individual sensors. Harmonizing sensor nodes into sophisticated computation and communication infrastructure, called wireless sensor network (WSN), may have strong impact on a wide variety of sensitive applications [1–4] such as military, scientific, industrial, health and home networks. However, due to the half-duplex property of the sensor radio and the broadcast nature of wireless medium, limited bandwidth remains a pressing issue for wireless sensor networks. The bandwidth problem is more serious for multi-hop wireless sensor networks (WSNs) due to interference between successive hops on the same path as well as that between neighboring paths. As a result, conventional single channel medium access protocol (MAC) protocol cannot adequately support the bandwidth requirements.

In the state-of-the-art research, significant attention has been paid to the design of throughput maximizing MAC (Media Access Control) protocols [5–11] that work well when one physical channel is used. However, due to the limited radio bandwidth in WSNs (e.g., 19.2 Kbps in MICA2 [12], 250 Kbps in MICAz [13] and Telos [14]), single channel MAC protocols further limit the higher demand for the bandwidth. Radio transceivers for wireless sensor networks are typically cheap devices offering low bandwidth communication only. When physical events in the real world trigger spontaneous communication in many nodes, the single communication channel is under heavy load and many messages are lost due to collisions. CSMA/CA schemes are well suited to spontaneous communication, but do not provide high channel utilization under heavy load. Therefore, another cost effective solution has drawn attention with the possibility to use multiple channels. The solution works for parallel data transmission based on the current WSN hardware, such as MICAz and Telos that provide multiple channels with single radio.

A number of multi-channel MAC protocols have been developed for general wireless networks [15–17] with single radio. Considering typical applications and capability of WSNs, these protocols are not suitable. Due to the small MAC layer packet size in WSN compared to general wireless networks, protocols such as [15–17] designed with RTS/CTS or 3-way handshake for channel/time negotiation provide significant control overhead for the constrained sensor nodes. Therefore, multi-channel MAC protocol for WSN should consider the minimum control overhead possible in negotiating the time/channel selection. Researchers have proposed few multi-channel MAC protocols [18–21] that exploit multiple channels to increase the network throughput in WSNs. However, these protocols suffer from high control overhead.

In this paper, we develop a schedule-based multi-channel MAC protocol for static wireless sensor networks. Our goal is to improve the network throughput using conflict free multi-channel scheduling. The approach is fully decentralized and efficient within the sensors’ localized scope. The scheme has been simulated to evaluate the effectiveness in terms of aggregate throughput, packet delivery ratio, end-to-end delay, and energy consumption.

The rest of this paper is organized as follows. Section 2 reviews the related works. Section 3 describes network model, assumptions, and problem statement. Section 4 presents the proposed scheme in details. Section 5 presents performance evaluation through simulations. Finally, Section 6 concludes this paper with summary and directions for future work.

## 2. Related Works

In the context of wireless sensor networks, there exist recent proposals that use the concept of multi-channel media access techniques to improve the network performance. Zhou *et al*. [18] recently introduced the MMSN multi-frequency MAC protocol especially designed for WSN. It is a slotted CSMA protocol and at the beginning of each timeslot nodes need to contend for the medium before they can transmit. MMSN assigns channels to the receivers. When a node intends to transmit a packet it has to listen for the incoming packets both on its own frequency and the destination’s frequency. A snooping mechanism is used to detect the packets on different frequencies, which makes the nodes to switch between channels frequently. MMSN uses a special broadcast channel for the broadcast traffic and the beginning of each timeslot is reserved for broadcasts. MMSN requires a dedicated broadcast channel.

The MC-LMAC protocol [21] uses a scheduled access where each node is granted a timeslot beforehand and uses this timeslot without contention. At the start of each timeslot, all the nodes are required to listen on a common channel in order to exchange control information. However, the protocol overhead is significantly high. TMCP [22] is a tree-based multi-channel protocol for data collection applications. The goal is to partition the network into multiple subtrees with minimizing the intra-tree interference. The protocol partitions the network into subtrees and assigns different channels to the nodes residing on different trees. TMCP is designed to support convergecast traffic and it is difficult to have successful broadcasts due to the partitions. Contention inside the branches is not resolved since the nodes communicate on the same channel.

There are many MAC protocol proposals which consider single-channel communication [5,7,8,10] in the domain of wireless sensor networks. These protocols perform to be good in single-channel scenarios where the primary design goal is energy efficiency [23], scalability and adaptability to changes [24].

There are single-channel MAC protocols that aim to provide high-throughput especially with scheduled communication such as Z-MAC [25], Burst-MAC [26]. While these protocols perform well in single-channel scenarios, parallel transmissions over multiple channels can further improve the throughput by eliminating the contention and interference on a single-channel.

Besides multi-channel communication there exist other methods to reduce the impact of interference such as transmission power control [27], creating minimum interference sink trees [28]. In a previous work [29] authors have investigated the impact of transmission power control on the network’s performance with a realistic setting and found that discrete and finite levels of adjustable transmission power on the radios may not completely eliminate the impact of interference.

In our proposed mechanism, data transmission and reception scheduling as well as actual data transmission are performed in a collision free manner. Unlike the existing protocols, we split a cycle time into three parts. In the first part, the beginning of a cycle, each node simply acquires the order in which each node will announce the data transmission/reception schedule. In the second part, nodes broadcast its schedule according to this order. Each node broadcasts its transmission schedule along with the scheduling information of its neighbor nodes only once. Finally, each node actually transmits the data packets according to the schedule announced in the second part of the cycle. Since, each node uses one broadcast to announce its scheduling information; 2-hop node(s) may not hear the schedule. To overcome this problem, we propose a unique solution by splitting the data transmission time slots into different groups, where each node transmits/receives actual data packet(s) in one of the groups. Each node calculates its group using the number of groups and the order. This makes the scheduling collision free.

## 3. Network Model, Assumption and Problem Statement

### 3.1. Network Model and Assumption

We consider a wireless sensor network (WSN) that monitors a vast terrain of interest via a large number of static sensor nodes and a data collection point called sink/base station. This WSN can be represented by an undirected graph *G* = (*V,E*), where *V* represents the set of all sensors in the network and *E* *⊂* *V × V* represents the set of communication links between any pair of nodes. There is one data collection point called Base Station (BS) in *V*. All traffic generated at sensors is destined for BS. Such a network is called many-to-one sensor network. The distance *d*(*i, j*) between nodes *i* and *j* is defined as the minimum number of edges needed to traverse to go from one to the other. From this definition, the topology of sensor network can be described by an *N × N* symmetric adjacency matrix *C*, which is defined as *C_ij_* = 1, if *d*(*i, j*) = 1; else *C_ij_* = 0.

We assume that every sensor node has a unique ID. Each node is equipped with a half-duplex transceiver; a node can either transmit or listen, but cannot do both simultaneously. A transceiver can be tuned to different channels (non-overlapping frequencies) and all channels have the same bandwidth. The sink (or base station) is a data collection center equipped with sufficient computation and storage capabilities while the sensors are battery-operated and are empowered with limited data processing engines. Nodes are time synchronized [30] to provide efficient broadcast support. The task of the sensors is to dynamically serve the need of data from the target area to the sink.

### 3.2. Problem Statement

The conflict relationship in the network can be described by an interference matrix *I_N×N_*, where if *d*(*i, j*) *≤* 2, *I_ij_* = 1; else *I_ij_* = 0. This conflict relationship (due to interference) leads to two conditions for parallel transmission to be successful: (1) node *i* and *j* can transmit data on the same channel at the same time if the communication distance *d*(*i, j*) is larger than 2, and (2) if the communication distance *d*(*i, j*) is less than or equal to 2, node *i* and *j* can transmit data at the same time on different channels.

To design a multi-channel MAC, usually a period of time is split into some equal intervals called timeslots. Each timeslot is designed to accommodate one or more packets to be transmitted and received between pairs of nodes in the network. Hence, the allocation of timeslots directly influences the network performance. Furthermore, proper channel/timeslot allocation also ensures collision-free communication when several transmissions run simultaneously. So, an efficient way of scheduling the channels/timeslots is required to maximize the network throughput and improve other performance issues such as delay, energy consumption, *etc*.

## 4. Proposed Multi-channel MAC Protocol

The goal of this section is to devise an efficient multi-channel MAC protocol that carefully schedules message transmissions so as to avoid collisions at the MAC layer, and thereby to utilize multiple channels to maximize parallel transmission among neighboring nodes. Our media access design is fully distributed and avoids multi-channel hidden terminal problem [16].

Our main concern is to devise a methodology so as to avoid the collisions among transmission of different sensor nodes. The key reason behind the collisions is the so called hidden terminal problem, which is caused when a sender is not aware of the transmission of another sender. Moreover, in many cases, senders may not even notice the collisions if they are out of the interference range. It is the receiver who actually faces the problems in receiving due to the collisions. Keeping this in mind, channel assignment in our protocol is made receiver-based. During the network initialization, receiving channels are assigned to the nodes for data reception and each node broadcasts its receiving channel to its neighbors. When a node wants to transmit data, it needs to switch to the receiver’s receiving channel.

In the proposed protocol, different time slots are assigned to different sender-receiver pairs and the use of multiple channels assures parallel transmissions between different sender-receiver pairs in the same time slot over different channels. The data transmission schedules in different channels in different time slots are done carefully avoiding collisions. When a receiver selects a channel as well as a data reception slot, it is aware of the other schedules that are already chosen by the other nodes within its interference range (typically the nodes within its two-hop distance).

In our protocol a cycle (time duration) consists of three parts: (1) Contention Period (CP)—to provide an ordering to the nodes, (2) Control Slot Window (CSW)—to perform the data transmission scheduling algorithm, and (3) Data Transfer Window (DTW)—where the actual data transmissions take place. However, once the data transmission slots are chosen (during CP and CSW), the nodes can use the schedule (repeating the DTW only) until any change is necessary due to topology changes (e.g., node failure, *etc*.). We describe our protocol in detail in the following subsections.

### 4.1. Cycle Structure

**Figure 1.**
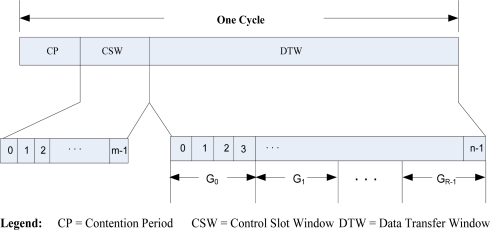
Cycle structure: a cycle is divided into contention period (CP), control slot window (CSW) and data transfer window (DTW). DTW is divided into R groups.

The structure of a cycle is shown in Figure 1. As stated earlier, one cycle (time duration) is divided into three parts, namely contention period (CP), a control slot window (CSW), and a data transfer window (DTW). Both the CSW and DTW are contention free periods (CFP). CSW is divided into *m* (0,1,2,…,m − 1)equal sized slots. Duration of a slot in CSW is set to the time for picking up the desired reception slots plus the transmission/reception time of a control message. Similarly, DTW is divided into *n* (0,1,2,…,n − 1) timeslots of equal length, and the duration of a slot in DTW is set to transmission/reception time of one or more data packets along with the ACKs. Timeslots in DTW is further categorized into *R*(*G*_0_, *G*_1_, *G*_2_, …, *G_R_*_−1_) groups with equal number of timeslots. The selection of the parameters *m*, *n* and *R* are discussed in later sections of this paper.

### 4.2. Scheduling Transmission

For the communication during CP at the beginning of a cycle, nodes use the broadcast mechanism used in 802.11 CSMA/CA. During this period, each of the nodes stays in a common channel and contends for a slot in the CSW. Each node obtains an order *s*(0≤*s*<*m)*, *i.e.*, the slot in CSW, by contending each other during CP (the parameter *m* is set to the maximum number of nodes that may fall within 1-hop neighborhood of a node, including itself). By doing so, Theorem 1 guarantees that no other node within the 2-hop communication distance of a node will have the same order.

**Theorem 1.** If every node within 1-hop neighbors of a node selects a distinct slot in the CSW, it ensures that no other node within its 2-hop neighbor will select the same slot.

**Proof** Suppose that two nodes *A* and *B* fall within the 2-hop neighborhood of each other, and they have selected the same slot *s* in the CSW. There must be another node *C*, which has node *A* and node *B* within its 1-hop neighborhood. This contradicts with the proposition.

During CSW, all nodes select timeslots/channels to be used during the following data transfer window. The nodes also tune to a common channel to broadcast their selections to others. The CSW is divided into *m* slots, which are allocated to the nodes according to the order *s* that they get during the CP. During its assigned slot in CSW, every node selects some empty slots (in DTW) in its receiving channel for data reception from the nodes that will send data to it. Allocation of such slots is done by every node that is likely to receive data from some other nodes. The selection can be done in a distributed manner according to Algorithm 1. Every node *node_r_* follows the Algorithm 1 during its slot *s* in CSW.

The data structures that are used in our algorithms are listed here:

*Channel*: stores transmission schedule of *n* slots in a channel

- *Sender*[*n*]
- *Receiver*[*n*]
- *Node*: stores information of a node:
- *recvChannel* /*receiving channel */
- *s* /* order in the CSW */
- *channel*[*nc*] /*nc = no of channels*/

In Algorithm 1, all the nodes having the same order *s* allocates there timeslots in DTW simultaneously. As the orders are 2-hop aware, there will be no chance of collisions among the simultaneous transmissions that are received by these nodes. After selecting the slots, a node updates and broadcasts its *channel* information containing its schedule along with that of the others available to it. Nodes within the transmission range update this information by overhearing this broadcast message. Upon receiving the message from a node *node_r_*, a transmitting node can know at which slot in DTW it should transmit data to *node_r_* and what channel to use.

**Algorithm 1:** AssignTransmissionSlots

*y* = *s* mod *R*;	/*select the group*/
*f =* min(0≤*i < m)*, where *i* mod *R* = *y*	
*Pos* = *s*/*R*;	/*Position in group*/
**for***k* = *Pos–*1 **down to***f***do**	
**if***node_r_* does not have slot allocation information of any node having order *k* in its 2-hop neighborhood **then**	
**break;**	
**end if**	
**end for**	
*NoInfo* = *k – f–* 1;	
*AssignSlots*(*node_i_*,*G_y_, NoInfo*);	
Broadcast *node_i_*.*Channel* to the one hop neighbors.	

A node *node_r_* picks a timeslot *slot* for a transmitting node *node_i_* in its receiving channel *ch* following Algorithm 2. Prior to selecting the timeslot, *node_r_* checks if either of *node_i_* and *node_r_* pre-exists in *slot*, or if *ch* is occupied by other transmission during *slot*. This confirms collision free scheduling.

In some cases, it may happen that a node *node_r_* does not have the information about the scheduling done by some of the other nodes in its 2-hop neighborhood having lower control slot orders in CSW. In such cases, it reserves some *SafetySpace*s in the beginning of the DTW, from which those nodes may have selected their *slot*s. *node_r_* tries to allocate form the other slots for itself. However, this may lead to reservation a large number of slots as *Safetyspace*, which may not be always feasible. To minimize this, our protocol partitions the DTW in *R* groups, *G_y_* (0 ≤ y ≤ *R*−1). A node can select its timeslot(s) from one of the *R* groups according to Equation 1:

(1) y=smodR

This grouping has two advantages. Firstly, since every node has to be aware only of the nodes in its respective group, the information overhead for a node is minimized. Secondly, it minimizes the amount of unavailable information necessary for a node in selecting channels as well as timeslots. This reduces the timeslot(s) to be reserved as *safetyspace*(s). Consequently, the reduction in *safetycpace*(s) reduces the required number of timeslots *n* in DTW, which in turn increases the throughput.

The DTW is grouped according to the ratio, *k*, of the interference and transmission range. In this paper we consider the value *k* = 2, and the DTW is grouped into *R* = *k* + 1.

**Algorithm 2:** AssignSlots(*node_i_*, *G_y_*, *NoInfo*)

*start* = group*Gsize;	/* *Gsize* = *total slots in a group* */
SafetySpace = NoInfo*ns;	/* *ns = maximum number of intended senders of a node* */
*start* = *start* + *SafetySpace*;	
*end* = *start* + *Gsize* – 1;	
**for** each receiving node *node_r_* of *node_i_***do**	
**for***slot* = *start***to***end***do**	
**if** neither *node_i_* nor *node_r_* has a previous entry in *slot***then**	
* ch* = *node_r_*.*recvChannel*;	
**if** there is no entry for channel *ch* in *slot*	**then**
* node_i_*.*Channel*[*ch*].*Sender*[*slot*] = *i*;	
* node_i_*.*Channel*[*ch*]. *Receiver* [*slot*] = *r*;	
**break**;	/* *Try the next receiving neighbor* */
**end if**	
**end if**	
**end for**	
**end for**	

To ensure that every node receives at least one timeslot in DTW, the number of timeslots *n* in DTW should be $\mathrm{ns}\left(\frac{m}{R}-1\right)+1$, where *ns* is the maximum number of senders of a receiving node, and the product term defines the maximum number of possible *safetyspace*s that a receiving node may have to reserve for the other nodes in its group (in the worst case). Note that, if such maximum number is used for *n*, it is much likely to have some empty (unused) timeslots in the DTW. However, as mentioned earlier, the need for leaving *safetyspace*s is not much high since the necessary information of other nodes in group are likely to be available in most cases. Hence, some compromises can also be made in determining *n*, which may cause some nodes to receiving no packets in a DTW. However, since the packets are transmitted to all possible receivers, paths to the sink are much likely to exist even if some nodes are avoided. Unlike the RTS/CTS (2-way handshake) or request-response-reservation (3-way handshake), our scheme involves only one broadcast message needed for each node to schedule their transmissions.

### 4.3. Scheduling Example

**Figure 2.**
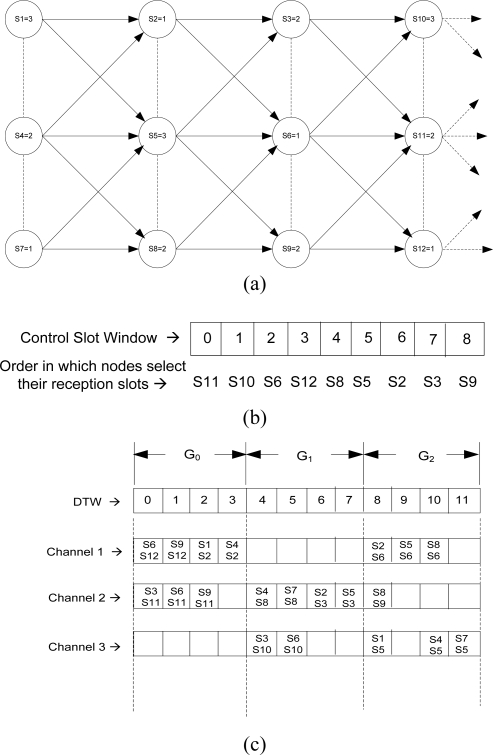
An example of transmission schedule for the proposed Multi-channel MAC protocol. **(a)** Topology with the node ID and receiving channel; **(b)** Control slot window and the order in which each node selects its reception schedule; **(c)** Allocated slots with three channels in a data transfer window where 12 timeslots are divided into three groups.

We describe the transmission scheduling algorithm with an example shown in Figure 2. Consider Figure 2(a) where each node is represented with their node ID and receiving channels, and each arrow shows its intended receivers to which it may transmit its data.

This example considers a small snapshot of nine nodes (S2, S3, S5, S6, S8, S9, S10, S11 and S12) from a large network that fall within 1-hop communication distance of S6. Assume that these nine nodes have got the slots in CSW according to the order shown in Figure 2(b) after contending during CP. As can be seen from Figure 2(b), S11 is the first node (*s* = 0) to select and announce its reception schedule, and it has 3 intended senders S3, S6 and S9. To determine the group, *y*, from which it selects its timeslots to receive data from its intended senders, S11 gets *y* = 0 according to Equation (1), which is the first group in DTW. Since it has three intended senders, it can select three slots form Channel 2 from the beginning of group 0 as shown in Figure 2(c). Then, it broadcasts its schedule so that its immediate neighbors can update this information.

In the similar manner, other nodes may select their timeslots/channels. Consider node S5 with control slot order 5. It will select the timeslots from group 2 according to Equation (1). Before selecting the slots, S5 checks the schedule of S6, which should have already chosen its slots. It knows, from the broadcast message of S6, which slots are selected from group 0 by S6, and accordingly S5 selects the slots 0 in Channel 3, as shown in Figure 2(c).

In the case when node nodes fall into the same group, but one node does not know the previous node’s schedule (*i.e.*, two nodes are not immediate neighbors and a node has not yet received the previous one’s broadcasted message via other nodes), the node can leave the timeslots (safety space) from the beginning of that group (since the previous node has already taken the slots from that group), and select the timeslots accordingly.

Nodes that are beyond 2-hops may get the same pattern of control slot order in CSW. For example, node S1, with same control slot order, may select its schedule at the same time as S10, and so on. In this way, only one node is receiving in a channel during a particular slot within 2-hops. Also the schedule allows parallel transmission within 2-hops with disjoint sets of source-destination pairs with different channels.

## 5. Performance Evaluation

The effectiveness of the proposed Multi-channel MAC protocol was judged through simulations. We consider three important performance metrics: aggregate throughput, packet delivery rate and average end-to-end packet delay as a function of number of channels. We compare our results with the CSMA, MMSN and MC-LMAC protocols.

In our simulation model, we assume a multi-hop network environment, where 100 nodes are uniformly randomly distributed over a square-shaped terrain. The sink is positioned on the midpoint of a boundary. We assume the network topology to be static and the radio range of all nodes are same. A free space propagation channel model is assumed with the capacity set to 250 Kbps. Packet lengths are 32 bytes for data packets. The maximum transmission range for a sensor node is assumed to be 40 m. Number of channels is varied from 1 to 10. Each node has a maximum of three intended receivers (immediate neighbors) towards the sink node.

**Figure 3.**
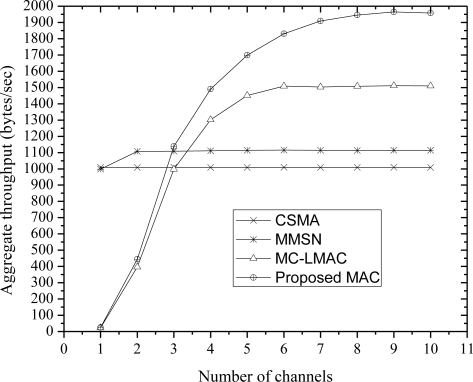
Aggregate throughput with different number of channels.

Figure 3 presents the results in terms of aggregate throughput. The aggregate throughput is shown (in number of bytes per second received by the sink node) as a function of number of channels. With the proposed MAC protocol, the maximum aggregate throughput at the sink node is 1,963 bytes/s. The results from CSMA, MMSN and MC-LMAC protocols are also presented to compare the performance. Figure 3 shows that aggregate throughput increases as the number of channels increases from 1 to 10 except CSMA where the number of channels is fixed to 1. A significant improvement is achieved using our proposed protocol compared to the CSMA, MMSN and MC-LMAC protocols. On the average, single channel CSMA achieves an aggregate throughput less than all the other protocols. Due to the high contention, the protocol fails to successfully allocate the medium to the nodes. Aggregate throughput with MMSN is observed to be limited and does not increase after six channels. This is due to the failure of the nodes around the sink to successfully sense the channel and prevent the collisions. MC-LMAC suffers from clashes that occur during the selection of the free timeslot(s) within two-hop nodes.

**Figure 4.**
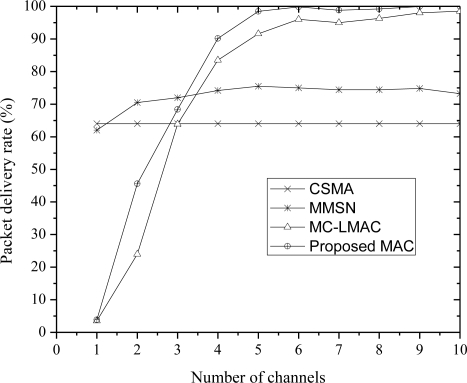
Packet delivery rate with different number of channels.

Figure 4 presents the results in terms of packet delivery rate which is the ratio between the number packets received by the sink and total number of packets generated by the nodes. The performance is better than both protocols and achieves to deliver more than 99% of the packets.

**Figure 5.**
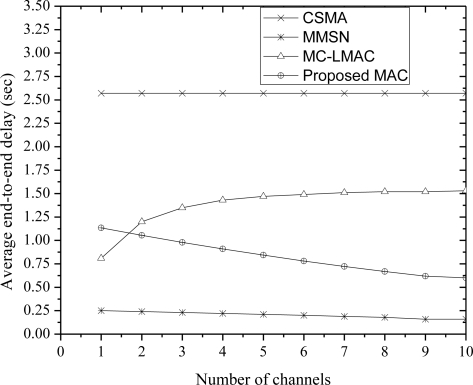
End-to-end packet delay with different number of channels.

Figure 5 presents the average end-to-end packet delay which is the time between the transmission of a packet at the source node and reception at the sink node. Our proposed protocol achieves much lower delay than the MC-LMAC protocol. Unlike the MC-LMAC, our protocol has decreasing end-to-end delay as the number of channels increases. This is because the average delay from source to the sink is influenced by the size of a frame in MC-LMAC protocol. Furthermore, decreasing the frame size would not reduce the delay since, the number of packets that can be delivered per timeslot will also decrease and the packets will be buffered to be transmitted later. CSMA experiences higher delay than all the protocols due to the exponential and higher number of backoffs due to the high contention.

**Figure 6.**
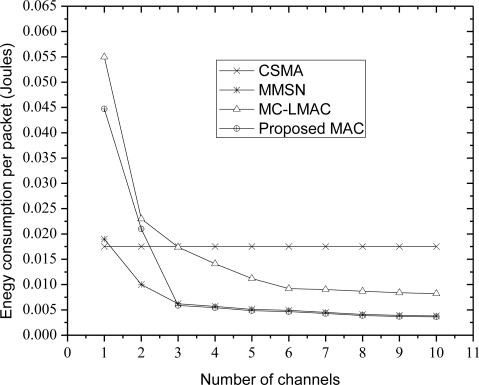
Energy consumption per successfully delivered packet with different numbers of channels.

Figure 6 shows the results in terms of energy-efficiency per successfully delivered packet. We consider both the energy spent to receive and transmit as well as the energy spent for relaying the packet towards the sink node. Energy spent per delivered packet is quite high with MC-LMAC when there is only a single channel. This is due to the very low delivery rate. As the number of channels increases, all the three protocols MC-LMAC and MMSN, and the proposed MAC spend much less energy than CSMA. Although MMSN has much less energy consumption compared to our proposed MAC in case of one and two channels, but our protocol is more energy efficient than all the protocols. This is because our protocol has much less collisions compared to the existing ones.

## 6. Conclusions

In this paper, we have developed a scheduled-based multi-channel MAC protocol for wireless sensor networks. Our protocol consists of a contention period to provide an ordering to the nodes, following which, in a control slot window, every receiving node selects some timeslots/channels from the data transfer window. The approach is fully decentralized and efficient within the sensors’ localized scope. The proposed protocol takes the advantage of control slot order along with the groups of data transmission/reception window to maximize the parallel transmission in a collision free manner. Also, each node needs only one broadcast message to advertise the schedule information that it has. Through simulations, it is shown that the proposed mechanism provides significant performance improvements in terms of aggregate throughput, packet delivery ratio, average delay, and energy consumption. As future works, we plan to run the experiment with different system loads and with different node densities. We also intend to delve into the performance issues for the mobile sink and multiple sinks.
